# Five Advances in Benign Pancreatic Surgery in the Last 50 Years

**DOI:** 10.1002/wjs.70369

**Published:** 2026-04-16

**Authors:** Stephan B. Dreyer, Rowan W. Parks

**Affiliations:** ^1^ Department of Surgery Royal Infirmary of Edinburgh Edinburgh UK

## 1 Introduction

Fifty years ago, pancreatic surgery was infamous for it's associated high morbidity and mortality rates with questionable patient benefits. The armamentarium of procedures available for benign pancreatic conditions were limited, almost entirely, to major resectional surgery such as Whipple's pancreaticoduodenectomy, distal pancreatectomy or total pancreatectomy. In the current era, pancreatic surgery and pancreatology are defined by significant technical advances to treat some of the most challenging conditions, with major improvements of outcomes in the management of benign pancreatic conditions. In the 1970's, post‐operative mortality rates exceeded 25% and thus the benefits were seen to be of limited value for patients with benign conditions.

Since then, there has been an evolution in surgical approaches to the pancreas, particularly for benign conditions, from maximum tolerated treatment to minimum effective intervention. This philosophy transcends what we believe are the five most significant milestones that define progress in the management of benign pancreatic pathology over the last half century.

### 1.1 The “Step‐Up” Approach for Infected Necrotizing Pancreatitis

There has been no greater advance in the management of benign pancreatic disease as in the minimally invasive management of necrotizing pancreatitis. For most of the 20th century, infected pancreatic necrosis was primarily treated with laparotomy, surgical debridement, continuous lavage and often required a laparostomy leading to high mortality rates (> 60%) and significant morbidity for almost all patients. A seminal study by Carter et al. described a technique for percutaneous necrosectomy that resulted in significantly less mortality (10%) and morbidity, particularly long term sequalae [Carter 2000] [1]. Using the mature tract formed by a radiologically placed drain, a nephroscope (or similar endoscope) was used to enter the retroperitoneum, debride the necrosum and place an irrigation catheter and drain [1]. Video assisted retroperitoneal debridement (VARD) evolved from this concept, but used a slightly different technique to gain access to the collection [2]. Most importantly, however, this proved to be a turning point in the philosophy of the management of infected pancreatic necrosis, from maximally invasive early surgery to minimally invasive, deliberately delayed interventions which were ultimately coined as the “Step‐Up approach” for management of pancreatic necrosis.

In 2010, the results of the PANTER trial confirmed this strategy to be superior to open necrosectomy by reducing major complications by 50% and by eliminating the requirement for necrosectomy in 35% of patients who required simple drainage alone [3]. The POINTER trial investigated whether immediate drainage, as soon as infection is suspected, or delayed drainage, when there is radiological evidence of the collection becoming “walled‐off” (usually 4–6 weeks after onset), was superior [4]. This trial demonstrated that delayed drainage resulted in 39% of patients requiring no drainage at all, and that they could be managed with antibiotics only [4]. This highlighted the significant progress made in reducing mortality and morbidity from long‐term external drains, laparostomies and early, maximally invasive drainage procedures.

### 1.2 Parenchymal Sparing Resections for Chronic Pancreatitis

For most of the 20th century, the surgical options for chronic pancreatitis were limited. The inflammatory mass that typically develops within the head of the pancreas leads to obstruction of the pancreatic duct, chronic pain and often obstructive jaundice. Pancreatoduodenectomy was the only surgical option for most of the last century, even though mortality was excessive in the early days of adoption. In 1958, Charles B. Puestow and William J. Gillesby described the Puestow procedure as a surgical solution for chronic relapsing pancreatitis [5]. Their original technique involved a longitudinal opening of the pancreatic duct, distal pancreatectomy and splenectomy, followed by a “telescoping” or invagination of the tail/body of the pancreas into a Roux‐en‐Y limb of the jejunum [5]. This was later modified by avoiding the distal pancreatectomy and splenectomy, instead opening the entire pancreatic duct and suturing the jejunum directly to the side of the pancreas (the so called lateral pancreatico‐jejunostomy) [6]. However, chronic pain and re‐intervention remained high after this procedure in those patients with an inflammatory mass in the head of pancreas, whose only other option was a pancreatoduodenectomy.

In the 1980's, Beger described the duodenum‐preserving pancreas head resection which involved resecting the pancreatic head whilst preserving the duodenum and maintaining biliary continuity [7]. Incorporating this, along with the original Puestow drainage philosophy, Charles Frey described a hybrid technique involving local resection or “coring” of the pancreatic head combined with a lateral pancreatojejunostomy [8]. These procedures, led to significant improvement in patient outcomes compared to pancreatoduodenectomy or the Puestow procedure, both in terms of peri‐operative outcomes and chronic pain, by addressing both the inflammatory mass in the pancreatic head and providing drainage of the distal pancreas. Since then, it has been shown that these procedures continue to provide good symptom relief, with better preservation of endocrine function than pancreatoduodenectomy or drainage only procedures [9, 10].

These advances have led to surgery becoming the gold standard for painful chronic pancreatitis in patients with a dilated pancreatic duct [11]. In patients with an inflammatory head mass, Frey's procedure is preferred, whilst if a dilated duct is present without a mass then a lateral drainage procedure provide excellent results compared to endoscopic management [11].

### 1.3 Endoscopic Management of Benign Pancreatic Disease

The evolution of pancreatic endoscopy from an invasive diagnostic tool to an all‐encompassing intervention, particularly for benign conditions, has been revolutionary. First described in 1968, endoscopic retrograde cholangio‐pancreatography, commonly known as ERCP, has become a crucial cornerstone in the management of pancreatic pathology. The refinement and development of endoscopic sphincterotomy, as well as pancreatic duct stents for pancreatic duct stones, strictures or leaks, was first described in 1974 and remains commonly used.

The success of ERCP, however, has arguably been surpassed by the development of endoscopic ultrasound (EUS). The advances in high resolution, high‐frequency transducers at the tip of the endoscope allows imaging of the pancreas on a level of granularity that exceeds even that of high‐resolution cross‐sectional imaging [12]. In conjunction with the development of the next generation of fine needle biopsy needles, EUS can provide extremely high diagnostic yield from lesional biopsy. The most exciting development is the opportunity for next generation sequencing from primary pancreatic biopsies or cyst fluid for molecular profiling in precision oncology approaches for pancreatic cancer, as well as distinguishing benign from malignant lesions with high accuracy [13, 14].

As well as being a highly accurate diagnostic tool, EUS has become a major cornerstone in the management of pancreatic walled‐off necrosis and pseudocysts. The development of lumen opposing metal stents (LAMS) in the early 2010's has been revolutionary in the minimally invasive management of these complications of acute pancreatitis. These “dumbbell‐shaped” stents allow the creation of a leak‐proof conduit between opposing hollow structures such as the stomach and peri‐pancreatic collections [15]. This allows for most pancreatic pseudocysts and necrotic collections to be managed endoscopically within the “step‐up” framework. This was investigated by the TENSION trial which compared percutaneous versus endoscopic drainage and found significant benefits in the endoscopic group [16]. Patients who underwent endoscopic drainage or necrosectomy, developed significantly less percutaneous pancreatic fistulae as well as having a shorter length of stay [16]. After a median follow up of 7 years, these benefits were shown to be maintained, leading to less incisional hernias compared to patients who underwent surgical drainage [17].

The huge success of LAMS in the management of pancreatic collections has resulted in ever expanding applications of the technique including cholecysto‐duodenostomy for acute cholecystitis, endoscopic gastro‐enterostomy for gastric outlet obstruction and choledocho‐duodenostomy for biliary strictures. Thus, complex endoscopy has become a crucial part of the armamentarium of the modern pancreatic clinician and is likely to continue to form the mainstay of intervention for benign pancreatic conditions in high volume centers.

### 1.4 Development and Maturation of Minimally Invasive Pancreatic Surgery

Despite the initial success of laparoscopy in the management of benign biliary disease, and even early description of laparoscopic liver surgery in the 1990s, pancreatic surgery became the “final frontier” for adopting laparoscopic surgery. The retroperitoneal location of the pancreas, the intimate proximity to major vascular structures and the high‐risk profile made adoption of laparoscopic pancreatic surgery challenging. Laparoscopic distal pancreatectomy, however, is now widely practiced and is accepted as the preferred surgical modality for benign pancreatic conditions including tumors and distal pancreatic duct strictures. The LEOPARD trial demonstrated improved short‐term outcomes in terms of functional recovery, length of stay and delayed gastric emptying for benign and low grade malignant lesions [18]. Laparoscopic pancreatoduodenectomy however, has not had the same widespread impact due to the complexity of the procedure and steep learning curve. The LEOPARD‐2 trial was stopped early due to higher mortality in the laparoscopic group, which significantly impacted the fervor for pursuing this and for further development of complex minimally invasive pancreatic surgery [19].

However, the advent of robotic assisted surgery has re‐invigorated the enthusiasm for developing minimally invasive pancreatoduodenectomy. The hugely improved optics and 3D visualization, along with wristed instruments have made huge advances in the ergonomic challenges associated with laparoscopic pancreatic surgery. This in turn, has allowed the development of minimally invasive drainage procedures for chronic pancreatis including Frey's procedure. Furthermore, in patients with benign lesions of the tail of pancreas, the robotic platforms allow higher rates of spleen‐preserving distal pancreatectomies, which may benefit patients in terms of their long‐term immune function. The integration of imaging technology, including ultrasound and fluorescence on robotic platforms allows for improved visualization of small benign lesions, particularly in their relationship to the pancreatic duct. This potentially facilitates enucleation procedures for small insulinomas.

### 1.5 Total Pancreatectomy and Islet Auto Transplantation for Chronic Pancreatitis

Total pancreatectomy and islet auto transplantation (TPIAT) has developed into a valuable and successful treatment option for highly selected patients with chronic pancreatitis. Patients with refractory pain that has failed medical or endoscopic management, and who are not ideal candidates for a surgical drainage procedure, can be considered. In addition to total pancreatectomy, which aims to relieve the debilitating pain of the condition, re‐infusion of viable islet cells aims to maintain endocrine function. TPIAT involves total pancreatectomy and is accompanied by retrieval and subsequent re‐infusion of viable islet cells to mitigate the associated brittle diabetes [20].

Despite its promise, TPIAT remains a rarely performed procedure, except in high volume centers. Clinical outcomes demonstrate that TPIAT provides significant and durable pain relief, with a substantial proportion of patients achieving opioid independence. Sutherland et al. reported results from 30 years' experience of treating over 400 patients [20]. All patients were on opiates prior to surgery and this reduced to only 40% at 3 years. They found that 84% of patients had significant improvement in pain, and a third were insulin independent [20]. The results were even more encouraging in children [21]. Reported rates of opiate independence range from 50%–80% at long‐term follow‐up in other studies [21, 22, 23]. Additionally, approximately 30%–40% of patients achieve insulin independence, while many others retain partial graft function, resulting in improved glycemic control compared with total pancreatectomy alone [20, 24].

Patient selection is critical to optimizing outcomes. TPIAT is most effective when performed earlier in the disease course, before extensive pancreatic fibrosis compromises islet yield and opiate dependence is established. Factors associated with better endocrine outcomes include higher islet cell mass, younger age, and absence of preoperative diabetes [22]. Conversely, patients with long‐standing disease or significant pancreatic calcification may have reduced islet viability. A major advance in this area would be to improve islet yield during the retrieval process. Novel techniques in islet isolation and preservation are coming to clinical practice, which may improve endocrine function following TPIAT [25]. In addition, major advances in stem cell biology has the potential to provide new options for these patients. Pluripotent stem cells can undergo chemically directed evolution into islet cells prior to implantation [26]. The benefit of this being that islet cells can be prepared, and potentially infused, prior to total pancreatectomy and result in improved endocrine function and diabetic control [26]. This has obvious potential for patients with type 1 diabetes mellitus, but also for patients with chronic pancreatitis requiring total pancreatectomy. This will add great promise as TPIAT remains a complex and resource‐intensive procedure, requiring specialized surgical expertise, islet isolation facilities, and multidisciplinary perioperative care.

## 2 Conclusion

Over the past 50 years, the management of benign pancreatic disease has evolved from high‐risk radical surgery to a multidisciplinary model centered on minimally invasive, organ‐preserving approaches. The development of the step‐up strategy for necrotizing pancreatitis has revolutionized care, significantly reducing the need for open surgery, while parenchymal‐sparing procedures in chronic pancreatitis have improved long‐term outcomes. Ongoing advances in endoscopic and robotic techniques, alongside progress in islet autotransplantation, continue to expand the therapeutic landscape.

Collectively, these innovations form the foundation of modern management, with future progress likely to further minimize morbidity and enhance patient outcomes through increasingly tailored, multidisciplinary care.

## Author Contributions

**Stephan B. Dreyer:** formal analysis, writing – original draft, conceptualization, investigation. **Rowan W. Parks:** conceptualization, supervision, writing – review and editing, formal analysis, investigation.

## Funding

The authors have nothing to report.

## Conflicts of Interest

The authors declare no conflicts of interest.

## Data Availability

Data sharing not applicable to this article as no datasets were generated or analysed during the current study.
